# Association between salt sensitivity and blood pressure variability in patients with essential hypertension and predictive value for cardiovascular events

**DOI:** 10.5830/CVJA-2022-048

**Published:** 2023-03-22

**Authors:** Biao Zhang, Dengyue Pan, Dexuan Zhao, Xiaohua Dai

**Affiliations:** Fuyang Second People’s Hospital, Fuyang, Anhui Province, China; Fuyang Sixth People’s Hospital, Fuyang, Anhui Province, China; First Affiliated Hospital of Anhui University of Chinese Medicine, Hefei, China

**Keywords:** salt sensitivity, blood pressure variability, essential hypertension

## Abstract

We aimed to explore the association between salt sensitivity and blood pressure variability in patients with essential hypertension. A total of 730 patients with essential hypertension treated from 2016 to 2019 were subjected to salt-sensitivity risk stratification according to 24-hour ambulatory blood pressure monitoring. Their clinical data were compared among groups with different grades of salt-sensitivity risk, and the association between salt sensitivity and blood pressure variability was analysed. The influencing factors for cardiovascular events in patients with essential hypertension were analysed through multivariate regression analysis, and their predictive value was detected using receiver operating characteristic (ROC) curves. Salt sensitivity was positively correlated with night-time and 24-hour systolic standard deviation and 24-hour systolic blood pressure coefficient of variation. Age ≥ 55 years, family history of cardiovascular diseases, high risk of salt sensitivity, night-time systolic standard deviation ≥ 14 mmHg, 24-hour systolic standard deviation ≥ 20 mmHg and 24-hour systolic blood pressure coefficient of variation ≥ 13.5% were all independent risk factors for cardiovascular diseases in patients with essential hypertension (p < 0.05). The area under the ROC curve of the prediction model was 0.837. There was a positive correlation between salt sensitivity and blood pressure variability, which has predictive value for cardiovascular events in patients with essential hypertension.

Essential hypertension is a complex disease caused by both genetic and environmental factors, and patients have a risk of damage to target organs such as the heart, brain and kidney, dominated by cardiovascular diseases.^1^ In the late 1970s, Luft et al.^2^ and Kawasaki et al.^3^ verified the existence of salt sensitivity in humans through long-term observation of blood pressure changes and degree of sodium retention under high-salt conditions.

Salt sensitivity refers to the different responses of an individual’s blood pressure to salt load or salt restriction.^4^ In the case of changes in sodium intake, blood pressure changes by at least 5–10% or 5 mmHg.^5^ Salt sensitivity is the genetic basis for the link between salt intake and hypertension, manifested as elevation of blood pressure due to relatively high salt intake, and differences and genetic tendencies among individuals, which is an intermediate genetic phenotype of essential hypertension.^6^

Patients with salt-sensitive hypertension suffer from early damage to target organs such as the heart, brain and kidney, often accompanied by impaired vascular endothelial function.^7^

Blood pressure varies with physiological and environmental changes. Blood pressure variability refers to fluctuation of blood pressure, expressed as the standard deviation of the change in blood pressure within a certain time period. Clinically, the individual’s fluctuation of blood pressure is generally exhibited using blood pressure curves, and the normal blood pressure curve displays double peaks and one valley.^8^

Blood pressure variability is primarily detected clinically by 24-hour ambulatory blood pressure monitoring, and it has been applied in the diagnosis and treatment of hypertension.^9^,^10^ The risk of cardiovascular diseases and target organ damage rises with increasing blood pressure variability, and blood pressure variability is a predictor for cardiovascular diseases.^11^

In the present study, the association between salt sensitivity and blood pressure variability in patients with essential hypertension was explored, and its predictive value for cardiovascular diseases was also investigated. Detecting salt sensitivity-related indices can help clinicians to timeously adjust the treatment regimens to reduce the incidence rate of adverse cardiovascular events.

## Methods

A total of 730 patients with essential hypertension treated in our hospital from 2016 to 2019 were selected as the subjects, and their baseline data were collected. Inclusion criteria were as follows: patients meeting the diagnostic criteria for essential hypertension, and those aged 18–75 years old. Exclusion criteria were as follows: patients with organic heart diseases, such as myocarditis, cardiomyopathy or valvular disease; those with severe liver or kidney insufficiency; patients with severe infections in the lungs or other sites; and those with severe malignancies or other autoimmune rheumatic diseases or connective tissue diseases.

This study was reviewed and approved by the ethics committee of the First Affiliated Hospital of Hebei North University. All the subjects were informed of this study and personally signed the informed consent.

Blood pressure was measured using an automatic 24-hour ambulatory blood pressure monitor (Spacelabs, USA). The monitor was fixed on the patient’s right upper arm by professionals, and the upper arm was kept relatively static during automatic measurement for 24 hours from 8:00 to 8:00 the next day (6:00 to 22:00 in the daytime, automatically measured every 30 minutes; 22:00 to 6:00 the next day in the night-time, automatically measured every 60 minutes).

The monitoring indices included 24-hour heart rate, daytime and night-time systolic blood pressure, daytime and night-time diastolic blood pressure, 24-hour mean systolic blood pressure and 24-hour mean diastolic blood pressure. The measurement results were valid if the valid data accounted for more than 80% throughout the day.

The blood pressure rhythm was calculated: (daytime mean blood pressure–night-time mean blood pressure)/daytime mean blood pressure × 100%.

The blood pressure rhythm of 10–20% and < 10% indicated dipper and non-dipper blood pressure, respectively. With blood pressure circadian rhythm and heart rate as evaluation indices, the subjects were divided into a low-risk group (24-hour heart rate ≤ 70 bpm, dipper blood pressure), a medium-risk group (24-hour heart rate < 70 bpm, non-dipper blood pressure; or 24-hour heart rate > 70 bpm, dipper blood pressure) and a high-risk group (24-hour heart rate ≥ 70 bpm, non-dipper blood pressure) according to the subjects’ 24-hour heart rate and whether blood pressure was dipper pattern or not.^12^

Fasting venous blood was drawn in the morning, centrifuged at 3 000 rpm for 10 minutes and stored at –20°C. A biochemical analyser was used to measure levels of fasting blood glucose, total cholesterol, triglycerides, highand low-density lipoprotein cholesterol, uric acid and serum creatinine through spectrophotometry. The electrocardiographic indices were monitored for 24 hours using a TLC5000 dynamic electrocardiograph (Shanghai Lixin Instrument Co, Ltd).

After the interference signals such as ectopic cardiac rhythm were eliminated, the standard deviation of the normal-to-normal intervals and standard deviation of the averages of normal-tonormal intervals in all standard deviation of sequential fiveminute normal-to-normal intervals were calculated using the computer system. Blood pressure variability was expressed as blood pressure standard deviation and coefficient of variation. Moreover, daytime systolic pressure standard deviation, nighttime systolic and diastolic standard deviation, 24-hour systolic and diastolic standard deviation, 24-hour systolic and diastolic blood pressure coefficient of variation, daytime systolic and diastolic blood pressure coefficient of variation, night-time systolic and diastolic blood pressure coefficient of variation were calculated.

The diagnostic criteria for essential hypertension were based on the Chinese Guidelines for the Diagnosis and Treatment of Hypertension 2013,^13^ which is daytime systolic arterial pressure ≥ 140 mmHg or/and diastolic arterial pressure ≥ 90 mmHg in three or more detections, and patients with secondary hypertension were excluded. Drinking was defined as alcohol consumption ≥ 50 ml/time, once or more per week on average for one year.

## Statistical analysis

IBM SPSS 19.0 software was used for statistical analysis. The normality test was conducted for the measurement of data. Normally distributed measurement data are expressed as mean ± standard deviation, while abnormally distributed measurement data are expressed as median. Comparison was made with the independent-samples t-test between two groups, and with one-way analysis of variance among the groups. The SNK test was conducted for pairwise comparison. Enumeration data were compared with the chi-squared test between two groups, and with the Kruskal–Wallis rank sum test among the groups. Spearman rank correlation analysis was performed between salt sensitivity and blood pressure variability, the risk factors for cardiovascular events in patients with essential hypertension were analysed through Cox multivariate regression analysis, and their predictive value for cardiovascular events was detected using receiver operating characteristic (ROC) curves. A p < 0.05 was considered statistically significant.

## Results

All of the 730 subjects were subjected to salt-sensitivity risk stratification according to 24-hour ambulatory blood pressure monitoring and blood pressure circadian rhythm. There were 141 cases (19.32%) in the low-risk group, 410 cases (56.16%) in the medium-risk group and 179 cases (24.52%) in the high-risk group. The baseline data were compared among the three groups. The results showed that among the three groups, the patient’s age and family history of cardiovascular diseases had significant differences (p < 0.05), but gender, body mass index, course of hypertension, history of smoking and drinking, and history of diabetes had no significant differences (p > 0.05) (Table 1).

**Table 1:** Baseline data of patients with different grades of salt-sensitivity risk

*Baseline data*	*Low-risk group (n=141)*	*Medium-risk group (n=410)*	*High-risk group = 179)*
Male/female	69/72	210/200	92/87
Age (year)	53.1 + 11.8	55.4 + 1 13.2	57.3 + 12.5a,b
Body mass index (kg/m²)	25.9 + 3.7	26.2 + 4.3	27.8 + 4.6
Course of hypertension (year)	10.5 + 3.1	9.6 + 4.7	9.3 + 5.9
History of smoking, n (%)	21 (14.89)	78 (19.02)	39 (21.79)
History of drinking, n (%)	18 (12.77)	66 (16.10)	27 (15.08)
History of diabetes, n (%)	28 (19.86)	103 (25.12)	37 (20.67)
Family history of cardiovascu- lar diseases, n (%)	21 (14.89)	62 (15.12)	58 (32.40) a,

a*p *< 0.05 vs low-risk group, b*p *< 0.05 vs medium-risk group.

The clinical detection indices were compared among the three groups. The results revealed that among groups with different grades of salt-sensitivity risk, low-density lipoprotein cholesterol and standard deviation of sequential five-minute normal-to-normal interval had significant differences (p < 0.05), while fasting blood glucose, total cholesterol, triglycerides, highdensity lipoprotein cholesterol, uric acid, serum creatinine and standard deviation of the normal-to-normal intervals had no significant differences (p > 0.05) (Table 2).

**Table 2:** Clinical indices of groups with different grades of salt-sensitivity risk

*Clinical indices*	*Low-risk group (n = 141)*	*Medium-risk group (n=410)*	*High-risk group (n=179)*
Fasting blood glucose (mmol/l)	6.02 + 1.06	6.13 + 1.78	6.07 + 1.35
Total cholesterol (mmol/l)	4.75 + 1.17	4.86 + 1.06	4.78 + 1.28
Triglyceride (mmol/l)	1.63 + 1.04	1.94 + 1.01	1.89 + 1.32
High-density lipoprotein cholesterol (mmol/l)	1.25 + 0.37	1.29 + 0.46	1.36 + 0.52
Low-density lipoprotein cholesterol (mmol/l)	2.87 + 1.05	3.12 + 1.43	3.54 + 1.79ab
Uric acid (umol/l)	327.65 + 83.14	349.28 + 91.36	346.81 + 87.25
Serum creatinine (umol/l)	64.58 + 19.27	69.13 + 22.75	65.59 + 20.16
Standard deviation of the normal-to-normal intervals (ms)	132.92 + 41.68	133.54 + 38.21	135.02 + 36.31
Standard deviation of sequential 5-min normal-to-normal interval (ms)	72.15 + 101.34	64.82 + 73.60	43.26 + 60.39 ,b

a*p *< 0.05 vs low-risk group, b*p *< 0.05 vs medium-risk group.

The ambulatory blood pressure-monitoring parameters and blood pressure variability were compared among the three groups. Significant differences were found in 24-hour heart rate, daytime systolic and diastolic blood pressure, night-time systolic and diastolic blood pressure, 24-hour mean systolic and diastolic blood pressure, daytime systolic and diastolic standard pressure deviation, night-time systolic standard pressure deviation,

24-hour systolic pressure standard deviation, and daytime systolic and diastolic blood pressure coefficient of variation (p < 0.05). No significant differences were detected in night-time diastolic pressure standard deviation, 24-hour diastolic pressure standard deviation, 24-hour systolic and diastolic blood pressure coefficient of variation, and night-time systolic and diastolic blood pressure coefficient of variation among the groups (p > 0.05) (Table 3).

**Table 3:** Ambulatory blood pressure monitoring parameters of groups with different grades of salt-sensitivity risk

*Parameters*	*Low-risk group (n=141)*	*Medium-risk group (n ==410)*	*High-risk group (n = 179)*
24-h heart rate (beat/min)	62.33 + 4.56	69.71 + 6.15	76.18 + 7.34ab
Daytime systolic blood pressure (mmHg)	122.37 + 9.15	136.89 + 10.52	144.96 + 13.78ab
Daytime diastolic blood pressure (mmHg)	74.23 + 6.71	83.12 + 11.68	87.45 + 8.96a,b
Night-time systolic blood pressure (mmHg)	110.34 + 7.58	129.67 + 10.37	142.17 + 13.02a,b,
Night-time diastolic blood pressure (mmHg)	66.07 + 8.36	76.91 + 10.19	89.85 + 11.23a, b
24-h mean systolic blood pressure (mmHg)	117.62 + 8.59	132.57 + 9.32ª	143.01 + 11.67a,b
24-h mean diastolic blood pressure (mmHg)	73.28 + 6.71	82.64 + 7.33	87.19 + 9.06a,b
Daytime systolic standard deviation (mmHg)	12.53 + 3.16	13.92 +	15.19 + 4.72a,b
Daytime diastolic pressure standard deviation (mmHg)	11.74 + 3.29	12.33 + 3.37	14.50 + 3.42ab
Night-time systolic standard deviation (mmHg)	11.67 + 3.28	13.55 + 5.64	14.95 + 3.67a,b
Night-time diastolic pressure standard deviation (mmHg)	9.08 + 3.41	9.65 + 4.12	10.33 + 6.07
24-h systolic standard deviation (mmHg)	15.27 + 4.96	19.36 + 4.15	23.69 + 5.02a,b
24-h diastolic pressure standard deviation (mmHg)	15.32 + 4.50	16.54 + 4.87	18.96 + 4.23
24-h systolic blood pressure coefficient of variation (%)	12.14 + 3.02	13.17 + 2.05	14.19 + 3.74
24-h diastolic blood pressure coefficient of variation (%)	17.11 + 4.03	18.15 + 6.02	19.18 + 5.01
Daytime systolic blood pressure coefficient of variation (%)	11.76 + 3.29	13.68 + 4.15	14.32 + 4.58a,b
Daytime diastolic blood pressure coefficient of variation (%)	17.65 + 5.34	18.19 + 7.22ª	19.75 + 6.08a,b
Night-time systolic blood pressure coefficient of variation (%)	11.25 + 4.37	10.76 + 4.12	11.58 + 4.62
Night-time diastolic blood pressure coefficient of variation (%)	14.58 + 6.35	14.86 + 5.99	15.02 + 6.92

a*p *< 0.05 vs low-risk group, b*p *< 0.05 vs medium-risk group.

According to Spearman rank correlation analysis, salt sensitivity of essential hypertension patients was positively correlated with night-time systolic standard deviation, 24-hour systolic standard deviation and 24-hour systolic blood pressure coefficient of variation (r = 0.827, 0.734 and 0.658, p < 0.05), but it had no significant correlations with other parameters (p > 0.05) (Table 4).

**Table 4:** Association between salt sensitivity and blood pressure variability

*Variables*	*r*	*p-value*
Daytime systolic standard deviation	0.053	0.142
Daytime diastolic pressure standard deviation	0.00	0.253
Night-time systolic standard deviation	0.827	0.019
Night-time diastolic pressure standard deviation	0.012	0.307
24-h systolic standard deviation	0.734	0.026
24-h diastolic pressure standard deviation	0.036	0.285
24-h systolic blood pressure coefficient of variation	0.658	0.045
24-h diastolic blood pressure coefficient of variation	0.047	0.192
Daytime systolic blood pressure coefficient of variation	0.004	0.791
Daytime diastolic blood pressure coefficient of variation	0.047	0.208
Night-time systolic blood pressure coefficient of variation	0.010	0.341
Night-time diastolic blood pressure coefficient of variation	0.002	0.892

During the one-year follow up, major cardiovas

**Table 5:** Cox multivariate linear regression analysis results of cardiovascular risk in patients with essential hypertension

*Independent variable*	*B*	*SE*	*Wald*	*p-value*	*HR (95% CI)*
Age	1.179	0.247	11.253	0.041	2.146 (1.603-3.588)
Family history of cardiovascular diseases	2.163	0.352	15.982	0.026	3.589 (1.960-5.026)
Salt-sensitivity risk stratification	1.687	0.309	13.927	0.033	3.015 (1.792-4.163)
Low-density lipoprotein cholesterol	0.129	0.358	0.743	0.596	1.153 (1.023-1.619)
Standard deviation of sequential 5-minute normal-to-normal interval	0.492	0.472	1.659	0.079	1.119 (0.936-1.258)
24-h heart rate	0.671	0.598	0.533	0.317	1.026 (0.985-1.358)
Night-time systolic standard deviation	1.586	0.636	16.742	0.013	4.569 (2.378-5.932)
24-h systolic standard deviation	1.231	0.821	15.683	0.025	3.651 (1.985-4.392)
24-h systolic blood pressure coefficient of variation	0.517	0.769	2.858	0.046	1.208 (1.106-2.536)

Age: ≥ 55 years: 1, < 55 years: 0; family history of cardiovascular diseases: yes: 1, no: 0; salt sensitivity: high risk: 1, medium and low risk: 0; low-density lipoprotein

cholesterol: ≥ 3.5 mmol/l: 1, < 3.5 mmol/l: 0; standard deviation of sequential five-minute normal-to-normal interval: < 50 ms: 1, ≥ 50 ms: 0; 24-h heart rate: < 62 beats/

min: 1, ≥ 62 beats/min: 0; night-time systolic standard deviation: ≥ 14 mmHg: 1, < 14 mmHg: 0; 24-h systolic standard deviation: ≥ 20 mmHg: 1, < 20 mmHg: 0; 24-h

systolic blood pressure coefficient of variation: ≥ 13.5%: 1, < 13.5%: 0.

cular events occurred in 105 patients. With the incidence of cardiovascular events as dependent variables, and age, family history of cardiovascular diseases, salt-sensitivity risk stratification, low-density lipoprotein cholesterol, standard deviation of sequential five-minute normal-to-normal interval, 24-hour heart rate, night-time systolic standard deviation, 24-hour systolic standard deviation and 24-hour systolic blood pressure coefficient of variation as independent variables, Cox multivariate linear regression analysis was performed. The results revealed that age ≥ 55 years, family history of cardiovascular diseases, a high risk of salt sensitivity, night-time systolic standard deviation ≥ 14 mmHg, 24-hour systolic standard deviation ≥ 20 mmHg and 24-hour systolic blood pressure coefficient of variation ≥ 13.5% were all independent risk factors for cardiovascular diseases (p < 0.05) (Table 5).

**Fig. 1:**
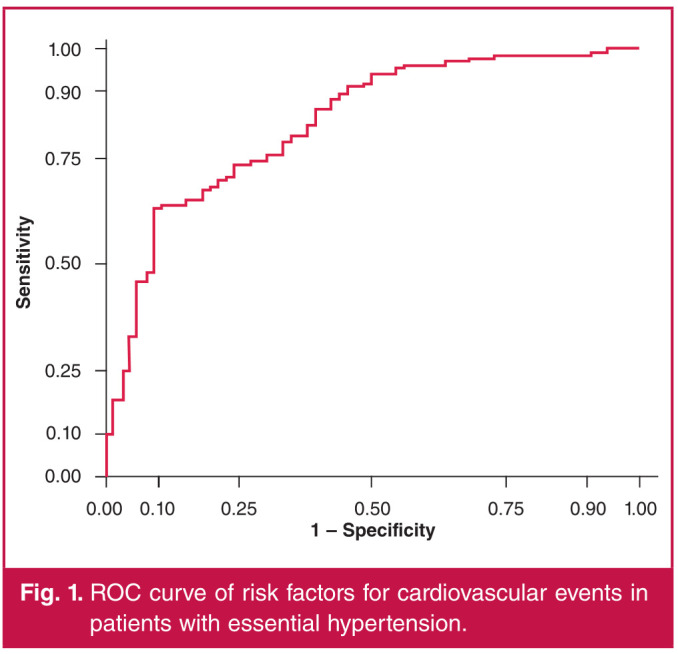
ROC curve of risk factors for cardiovascular events in patients with essential hypertension.

ROC curve analysis was conducted on the predicted results of the regression model for cardiovascular events and the actual results. The area under the ROC curve, sensitivity, specificity and Youden index were 0.837, 0.743, 0.899 and 0.859, respectively (Fig. 1).

## Discussion

Hypertension is the first risk factor for cerebral–cardiovascular diseases, and there are more than 200 million patients with hypertension in China.^14^ With the social development and changes in lifestyle in recent years, the incidence of hypertension and its related risk factors have increased, and how to effectively control hypertension has become an important public health issue. A high-salt diet is a risk factor for hypertension, stroke and cardiovascular diseases.

The association between salt intake and blood pressure variability in 605 patients with hypertension in Ningxia has been studied.^15^ The results showed that salt intake is an influencing factor for blood pressure variability in hypertensive patients, and reasonable salt restriction is important for controlling both blood pressure and blood pressure variability of patients. Salt sensitivity is susceptible to high salt intake. Jin et al.^16^ explored the correlation between salt sensitivity and the metabolic syndrome in Gannan Tibetan populations, and found that the detection rate of the metabolic syndrome was higher in salt-sensitive hypertensive and non-hypertensive populations, but the correlation remains to be further confirmed.

Ambulatory blood pressure monitoring is a simple, non-invasive examination that can display the patient’s blood pressure at different time points, and also provide the patient’s blood pressure circadian rhythm, blood pressure variability and morning peak blood pressure. The close associations of ambulatory blood pressure monitoring parameters with cardiovascular events and mortality have been confirmed.^17^

Ambulatory blood pressure monitoring can reflect the 24-hour ambulatory blood pressure, while blood pressure variability can reflect the influence of cardiovascular autonomic nerves on haemodynamics, which is considered a predictor for cardiovascular diseases independent of mean blood pressure.^18^,^19^

Cardiovascular events in hypertensive patients are not only related to blood pressure control level, but also closely related to blood pressure variability. The increase in blood pressure variability may lead to target-organ damage in hypertensive patients and its changes have attracted increasing attention in clinical treatment of hypertension.^20^

Wu et al.^21^ conducted screening for cardiovascular diseases among residents in six districts/counties in three towns and three villages in Liaoning Province, and explored the influencing factors for cardiovascular diseases. They found that the high-risk rate of cardiovascular diseases increased with age. In another study,^22^ cohort analysis was conducted on the clinical data of 985 patients with essential hypertension, and the results showed that 15 factors, including family history of cardiovascular diseases, were associated with major cardiovascular events. Cardiovascular diseases have a certain genetic tendency, and inquiring about the family history of cardiovascular diseases is helpful for predicting the risk of cardiovascular diseases in patients with essential hypertension.

The clinical indices of 382 patients with essential hypertension were analysed in the literature, and the levels of angiotensin and aldosterone were compared among patients with different grades of salt-sensitivity risk. The results revealed that the high-risk group had an increased level of aldosterone and a decreased level of angiotensin, further worsening target-organ damage in hypertensive patients.^23^ Moreover, 1 277 patients with essential hypertension were subjected to cardiovascular risk stratification, and ambulatory blood pressure monitoring parameters were compared in different groups. It was found that ambulatory blood pressure-monitoring parameters were correlated with the cardiovascular risk stratification of patients with essential hypertension, and four parameters, including night-time systolic standard deviation, were helpful for cardiovascular risk stratification of patients with essential hypertension.^24^

Wang et al.^25^ divided, by detecting 24-hour heart rate and blood pressure circadian rhythm, 315 patients with essential hypertension into low-, medium- and high-risk groups and compared the biochemical test results and ambulatory blood pressure monitoring parameters through univariate, multivariate and correlation analyses, with cervical–femoral pulse conduction velocity as the assessment index for vascular injury. They found that there was a positive correlation between 24-hour systolic standard deviation and cervical–femoral pulse conduction velocity. In a prospective study on 120 dialysis patients, Wang et al.^26^ explored the relationship between blood pressure variability and cardiovascular diseases, and confirmed that high 24-hour systolic blood pressure coefficient of variation was an independent influencing factor for cardiovascular death in dialysis patients.

In our study, the clinical data were collected and related indices were detected among 730 patients with essential hypertension, followed by salt-sensitivity risk stratification based on 24-hour heart rate. Then the basic data, laboratory indices, ambulatory blood pressure-monitoring parameters and blood pressure variability were compared among groups with different grades of salt-sensitivity risk. The results showed that age, family history of cardiovascular diseases, low-density lipoprotein cholesterol, standard deviation of sequential five-minute normal-to-normal interval, 24-hour heart rate, daytime systolic and diastolic blood pressure, night-time systolic and diastolic blood pressure, 24-hour mean systolic and diastolic blood pressure, daytime systolic and diastolic pressure standard deviation, night-time systolic standard deviation, 24-hour systolic standard deviation, and daytime systolic and diatolic blood pressure coefficient of variation had significant differences among the groups with different grades of salt-sensitivity risk. According to correlation analysis, salt sensitivity was positively correlated with night-time systolic standard deviation, 24-hour systolic standard deviation and 24-hour systolic blood pressure coefficient of variation in patients with essential hypertension.

It has been reported^27^ that patients with salt-sensitive essential hypertension have a higher risk of cardiovascular diseases and death. In the present study, age, family history of cardiovascular diseases, salt-sensitivity risk stratification, low-density lipoprotein cholesterol, standard deviation of sequential fiveminute normal-to-normal interval, 24-hour heart rate, nighttime systolic standard deviation, 24-hour systolic standard deviation and 24-hour systolic blood pressure coefficient of variation were incorporated into the Cox multivariate regression analysis model, so as to analyse the risk of cardiovascular events in patients with essential hypertension. The results manifested that age ≥ 55 years, family history of cardiovascular diseases, a high risk of salt sensitivity, night-time systolic standard deviation ≥ 14 mmHg, 24-hour systolic standard deviation ≥ 20 mmHg and 24-hour systolic blood pressure coefficient of variation ≥ 13.5% were risk factors for cardiovascular events. According to the ROC curve analysis of the above factors in predicting the risk of cardiovascular events in patients with essential hypertension, the area under the curve was 0.837, suggesting a good predictive effect.

However, this study has limitations. The correlation between salt sensitivity and blood pressure variability in essential hypertension patients and its predictive value for hypertension complicated with cardiovascular events were only preliminarily analysed. Besides, the effects of dietary habits, body weight or metabolism of the included patients were not considered. Hence, the results may be biased.

## Conclusion

There was a positive correlation between salt-sensitivity risk stratification and blood pressure variability, which had higher predictive value for cardiovascular events in patients with essential hypertension. 
